# Researches and Observations on the Causes of Scrofulous Diseases

**Published:** 1844-10

**Authors:** 


### Art. XVI.

*Recherches et Observations sur les Causes des Maladies Scrofuleuses.* Par J. G. A. Lugol, Médecin de l'Hôpital Saint-Louis, &c.—*Paris,* 1844.

*Researches and Observations on the Causes of Scrofulous Diseases.* By J. G. A. Lugol, &c.—*Paris,* 1844. 8vo, pp. 372.

The name and reputation of M. Lugol, and the extensive opportunities of observing disease which he has enjoyed, from his long connexion with the Hôpital St. Louis, led us, on the first announcement of the work before us, to hope that we should at length find, what has hitherto been a great desideratum in medical literature—a really good and comprehensive work on scrofula. The following pages, in which we shall endeavour to present our readers with a full, though condensed, analysis of the author's opinions, will show to what extent these expectations have been realized.

The work is divided into three parts. In the First, ***hereditary transmission of the disease*** is viewed under a variety of aspects; in the Second, the so-called *pathological causes* of scrofula are considered; and the Third treats of *external causes.* But throughout the entire volume the same one principle is constantly enunciated, viz. that scrofula is *always* hereditary; or, in the words of the motto which stands on the title-page, "*La santé des enfants tire son origine de la santé des parents.*"

We pass over the general observations contained in the preface and introduction, merely premising that M. Lugol agrees with most pathologists in regarding tubercle as the anatomical pathognomonic sign of scrofula; and that, in his opinion, this substance has the same origin and mode of formation as all the organs of the body; that it is itself a sort of organ which has its own particular life, as the liver and the spleen have theirs; that, like them, it is spontaneously developed; that it is a pathological production, which essentially modifies all the organic elements, and consequently their functions, and that it impresses upon the subjects affected by it a peculiar constitution, the tubercular—a constitution from which are subsequently derived the tubercles which may involve all the tissues, and an infinite number of diseases improperly called scrofulous. (Preface, pp. 5-6.) We trust our readers will fully comprehend all points of this profession of belief.

I. OF THE HEREDITARY ORIGIN OF SCROFULOUS DISEASES. This origin is recognized by two principal characters: 1, the commonness of the disease in any family; and 2, the great mortality which it occasions there.

The first is one of the most striking circumstances which meets an observer in studying scrofulous diseases; and even where the affection is not openly developed in all the members, he will notice that they all bear the external signs of a peculiar constitution, the most prominent features of which are here described.

The general conformation of the body is not good; the trunk and the extremities are not well proportioned; the head is too large, and, in general, the joints also. The median line is often not in the centre, and there is not rarely an incompleteness of union there, giving rise to separation of the linea alba and hare-lip, either simple or complicated with fissure of the palate. Some children are pigeon-breasted, the ribs being twisted and the sternum projecting upwards and forwards, so that the antero-posterior diameter of the chest exceeds the transverse. This deformity is sometimes gradually modified between the eighth and twelfth year, or at the time of puberty; but it more commonly persists, and is then a proof that the scrofulous taint is deeply rooted.

Scrofulous subjects are generally of small stature, but occasionally the direct opposite is the case; the teeth, both of the first and second dentition, but especially the latter, make their appearance slowly; they are generally of a dark colour, are prone to decay, and very friable. The spongy tissue of the bones is unduly developed when compared with their compact tissue and the soft parts. In the female pelvis this condition of parts often gives rise to serious difficulties in parturition.

The primæ viæ are in a state of atony, so that many scrofulous children never experience hunger. In M. Lugol's opinion, the cause of this is a catarrhal condition of the mucous membrane, precisely resembling that which in other parts occasions ophthalmia, otitis, bronchitis, &c., and which must be remedied before we can hope for any success in treating the general affection. In a few exceptional cases the appetite is voracious, but still the body is not well nourished. This state of the digestive canal is generally accompanied with habitual paleness of the face, a frequent precursory sign of pulmonary tubercles; with a dark circle round the eyes, fetid breath, and itchiness of the nose, giving rise to the supposition of the existence of worms. The excrementitial functions are irregular, constipation alternating with diarrhea.

The skin and cellular tissue are either abnormally delicate, or affected with an indurated hypertrophy. In many cases the skin is dry, and covered with patches of lichen or prurigo. There is a general deficiency of transpiration, coinciding with partial acescent perspirations of the feet, hands, and axillæ.

In infancy the face has an unnaturally aged look; but as years advance this passes off, and the adult subject appears much younger than he really is.

In many cases there is an extreme dislike to all exertion, and the carriage is very bad. The patients complain much of lassitude, which is not relieved but rather augmented by rest, for they feel more weary in the morning than during the course of the day. It is at this time also that the swelling of the upper lip is most strongly marked, and that ophthalmiæ are most painful. The latter circumstance is probably occasioned by the *sudden* transition from darkness to light, not by the mere influence of the light itself. This fact should be borne in mind, for M. Lugol has frequently observed that, excepting in a few instances of photophobia, much benefit is gained, in the treatment of scrofulous ophthalmia, by avoiding dark chambers, and allowing the eye to receive the impression of light.

Our author here indulges in a digression, into which it will be necessary that we follow him for a short space. He remarks, that it was from observing the general apathy of scrofulous subjects, and the injurious consequences which flowed from indulgence in this respect, that he was induced sixteen years ago to conjoin exercise with the iodine-treatment of white swellings of the lower extremities. Since that time, more than 300 cases have been thus treated, and, as he affirms, with good effect. "Why then, he asks, has not this treatment become general? Why? Because white swellings are still regarded as diseases of local origin, when they are merely the consequences of the tubercular predisposition." (p. 21.)

For ourselves, at the risk of being thought a century behind-hand in therapeutic skill, we must express a hope that this practice will not become general until far more solid reasons in its favour can be adduced, than the mere vague assertion thus laid before us. When it shall have been proved, on close and rigid scrutiny of sufficiently extensive trials, that *gelatinous degeneration of the synovial membrane*, *ulceration of the cartilages*, and *caries of the bones*, all of which, and more, are included under that most comprehensive and indefinite term "white swelling," when, we say, it shall have been proved that these various conditions, accurately diagnosed, are most speedily and effectually remedied by motion, then, and not till then, will we counsel our readers to yield their judgments captive to

M. Lugol, and set their patients promenading through the wards. But to return.

A certain degree of bodily activity is not incompatible with the scrofulous diathesis, but its effects, instead of favoring the growth of the body, rather act injuriously upon it, the system being unable to repair the waste.

The organs of generation are usually slowly developed. In the male, one testis frequently remains in the abdomen—more rarely both. In the female, menstruation is tardily established, and is seldom normal. Dysmenorrhea is very common. The mammary glands and ovaries are equally slow in their growth. But there are exceptions, as every one knows, to this rule.

In many cases, but not so commonly as is often supposed, the face shows evident marks of the constitutional taint, in the tumefaction of the upper lip, of the alæ nasi, the eyelids, especially their free border, and the lobule of the ears. These partial indurations, when they exist, are most significant. In the swollen upper lip M. Lugol states that he has recognized the first invasion of pulmonary tubercle; we suspect it was merely the predisposition, not the actual disease, that was thus revealed.

M. Lugol denies the frequency of *embonpoint* in scrofulous females. We need not say how deceptive is the appearance, when it is manifested—nor how easily a little attention will detect its true nature.

According to our author's experience few scrofulous persons have light hair; in more than half the hair is black, and among the rest a dark chesnut is the most usual colour. The same remark applies to the eyes and the skin, which are both most frequently of a dark tint. In our own country, the opposite is most commonly observed.

All these signs are rarely united in the same individual, their combinations are infinitely varied, and sometimes they are so slightly pronounced that a sharp eye is needed for their detection. Occasionally, too, some members of a decidedly scrofulous family, will pass through the greater portion of their life without exhibiting any marks of their hereditary taint, but at last, at an advanced age, fall victims to the disease, the germs of which they have borne within them, from birth: of this M. Lugol narrates some striking examples. (Vide p. 36 et seq.)

Lastly, we may observe, that, as a general rule, (but to this, though they are unnoticed by our author, there are numerous remarkable exceptions,) the intellectual faculties are below par. There may be some vivacity of mind, or even in youth precocity of intelligence, but there is a want of energy, of steadfastness, and power of concentrating the thoughts, which renders the unhappy inheritors of this temperament incapable of continuous exertion, and therefore of great deeds. In the words of the writer, "they experience no sustained activity of the physical desires, of the intellectual faculties, or of the moral sentiments; they have nothing normal, nothing powerful, nothing durable. All the phases of their existence fall short of their end; they have neither puberty nor manhood; the obstacles to their growth never cease to act; their physical and intellectual development remain unaccomplished."

In reference to the *general existence* of scrofula in any family, M. Lugol affirms, that if one of the children exhibits signs of the disease, we may, even without seeing the others, confidently assert that they also have the same malady. For, he remarks, if there be one thing impossible in pathology, it is that a tuberculous child should have brothers and sisters who are not so. Should any of our readers be inclined to doubt the correctness of this sweeping assertion, we beg to inform them that a very little thing will sign the condemnation of any individual in M. Lugol's eyes: it is quite enough for him if there be a somewhat troublesome ophthalmia or coryza, if the person be of rather stunted growth, or even if his spinal column slightly deviate from the perpendicular.* Apply these rules to all around us, and how many would remain untainted?

Setting aside, however, these exaggerations, which are perhaps natural to one who has made any particular disease his special study, the main fact is unquestionably true, and is a striking feature in the history of scrofulous diseases. We must refer to the work itself for many interesting examples; some of which are also amusingly illustrative of the hearty zeal with which the writer hunts out every token of hereditary taint.

We turn to a more melancholy picture—the *mortality* in scrofulous families. This is often frightful. Half the number of scrofulous children die during the first years of their life. We often see families in which not more than one or two children remain out of six or eight, or even a larger number: in many cases, none survive. M. Lugol is correct in affirming that such results as these can only originate from hereditary influence; external causes would scarcely effect such waste of life, and certainly would not work such mischief by inducing scrofulous diseases *alone.*

The *second section* in this chapter is occupied with the narration of numerous examples of the simultaneous manifestation of scrofula in the different branches originating from the same family stock; but on this point we need not delay, as our readers must have often witnessed parallel instances.

In the *third section* we are presented with another proof of hereditary transmission, in the health of the offspring of different marriages. Thus, if a man of sound constitution marry a scrofulous woman, he will have scrofulous children by her. Should he afterwards have a family by another, in good health, these will be free from taint, and vice versâ. Or, again, if a scrofulous individual be twice married, all the progeny will inherit the disease, whatever be the condition of the other partner. We suspect that to this rule also exceptions are not uncommon.

Having thus shown, that when scrofula exists in a family, it generally attacks all the children, and proves fatal to a large number during the early years of their existence, our author next proceeds to search for the origin of this hereditary taint, by examining into *the health of the parents who produce scrofulous children.* And here he remarks that he is far from affirming that all scrofulous persons are descended from parents who are themselves the subjects of the same disease: he believes that there are many causes by which a previously sound individual's health may be so effectually lowered, that his progeny will be born inheritors of this sad malady. In prosecuting our inquiries, therefore, it will be

* We should mention here, that throughout the volume *rachïtis* is spoken of as a scrofulous disease; and that very frequently the presence of *scirrhus* is appealed to as evidence of the strumous taint. The same remark will apply to *bronchocele* and *cretinism,* both of which are traced, we believe erroneously, to the same general cause.

necessary to examine the subject under two aspects: first, as regards the *original*, and, secondly, as regards the *acquired* health of the parents.

1. *Of the original health of parents who produce scrofulous children.* When the children's disease depends upon the original health of the parents, the latter are always scrofulous or tubercular. In most instances the external manifestations are sufficiently evident, and M. Lugol narrates a number of interesting cases, in which the disease was repeated in the children, under precisely the same form as it existed in the parents. He has frequently traced the contamination through three generations, and observes that when this is the case, the members of the last generally die almost at birth. He remarks also that he has never met with an instance in which the law of transmission has failed, when the father has been at fault, but has noticed a few rare exceptions when the taint was confined to the mother. This appears to us somewhat at variance with the dogmatic assertions of other parts of the work, though we make no question of its truth.

The next article touches upon a subject of the deepest interest and importance—the connexion between scrofula and phthisis. M. Lugol believes them to be identical; and laments grievously and, as we think, without just reason, that the profession should so generally regard the latter disease as a mere local affection, arising from inflammation. We are much mistaken if this be the *common* opinion; and assuredly Laennec is not, as the author insinuates, guilty of having either originated or built it up. In truth, he directly argues against it. "A multitude of facts," he says, "prove that the development of tubercles is the result of a *general condition of the body;* that it takes place without previous inflammation; and that when inflammation coincides with tuberculous affection, it is most frequently posterior to it in its origin." (Forbes's Laennec, 3d edit. p. 303.) But to proceed. M. Lugol enunciates three propositions: 1, that scrofula has most frequently a tuberculous origin; 2, that these two diseases generally coexist in the same family; and, 3, that all scrofulous persons are the subjects of pulmonary tubercles: these he seeks to establish in the following manner.

More than half the number of scrofulous subjects are descended from phthisical persons. Scrofula, under all its various forms of diseased cervical and mesenteric glands, white swellings, caries, ophthalmia, bronchitis, intestinal worms, hydrocephalus, &c. rages in a family for no other reason than that one of the parents has pulmonary tubercles. And how is this proved? Simply from the fact, that in two wards containing eighty-four beds, M. Lugol has commonly ascertained from more than half the patients that their parents were phthisical. How far these patients were accurately informed regarding the disease of which they spoke, we know not; but we can see that the inquirer was easily satisfied, for he remarks: "In most of these cases, in fact, we were told that the parents had been of a feeble constitution, and that they died young: two circumstances from which it is extremely probable that they died of pulmonary consumption." (p. 92.) We leave our readers to decide upon the philosophical caution displayed in this passage, and would beg them to contrast with it the words employed by his countryman, M. Louis, in referring to a parallel investigation.*

* Louis; Researches on Phthisis—Sydenham Society's edition, p. 485.

In support of the *second* proposition, he narrates several cases in which the fact was observed.

In regard to the *third*, he says: Phthisis is the natural death of scrofulous persons; it might even be affirmed that they die in no other way. The subcutaneous tubercles, ophthalmiæ, ulcers of the skin, caries of the bones, &c. only terminate fatally after the lungs have become tuberculous. This is a bold assertion—one that should be made only on the strongest grounds; but the sole confirmation with which we are favoured is the narration of two or three cases, and the statement that in *nearly all* the autopsies of scrofulous patients at St. Louis, tubercles have been found in the lungs. The exceptional instances are quickly dismissed, with the satisfactory remark, that when these morbid products are not discovered—the predisposition existing—it is because the time for their invasion had not yet arrived! (p. 96.) We are, however, promised a special work on tubercle, in which, perhaps, better reasons may be adduced.

It is not possible in every case to detect, at the first glance, the evidences of constitutional taint in the parents of scrofulous persons; and many, therefore, from confining their inquiries to a few superficial questions, have fallen into the error of attributing to external causes an influence which they did not really possess. The self-love of parents is also extremely apt to lead the investigator astray; and in numerous instances, even when his suspicions are most fully aroused, he will find it no easy matter to bring them to confess, that they themselves, or any of their family, have been previously afflicted with so hated and terrible a malady. It is necessary to bear these facts in mind; and we would direct the attention of our readers to the two next articles of the work before us, which contain some interesting examples of the propagation of scrofula by individuals who had suffered from the disease in youth, though at the time of the birth of their children they were in good health; or who had themselves never been affected, but merely belonged to a family, other members of which were scrofulous. These cases are also deserving of notice, because they exhibit in a very favorable light the practical acumen which the author frequently displays.

2. *On the acquired health of parents who produce scrofulous children.* Healthy persons have a healthy progeny. But there are many circumstances which, by their deteriorating influence upon the constitution, render an individual incapable of generating sound descendants. In the foremost rank of these M. Lugol places *syphilis.* He is no believer in the identity of the two diseases, though he recognizes their many points of resemblance; nor in the *general* origin of scrofula from the syphilitic taint; but he has frequently observed instances in which no other cause could be assigned, and which he therefore attributes to this. From among the few cases narrated we select one, as the most striking illustration. The youngest of a family of three children was scrofulous, the others being in perfect health. Their father had led a regular life, and enjoyed good health, until after the birth of the two eldest. He subsequently acquired disorderly habits, and contracted syphilis, with which disease he infected his wife. The child born under these circumstances presented evident marks of scrofula. Our author believes that it is the syphilitic cachexia alone that can produce this effect: primary syphilis can only propagate itself.

*Venereal excesses* are often followed by the same result. This M. Lugol attributes to the fact, that the seminal fluid is ejaculated immediately after its secretion, and without having been fully elaborated in the vesiculæ seminales.

We can merely indicate the other causes enumerated. They are, too early and too late marriages, disproportion of ages, want of relative physical force in the father, paralysis, epilepsy, insanity, &c. On many of these the evidence adduced is anything but satisfactory, and the reasoning most loose. We refer especially to one case, that of a young lady who exhibited signs of scrofula, after suffering from a mucous fever. M. Lugol totally rejects the idea that this fever had anything to do with the subsequent disease, though the only other cause he can assign is, that the father, a healthy man, was of a modest complexion, appeared feeble when compared with the "rich, abundant energetic organization of his wife!" (p. 142.)

In the next chapter, which is little more than an appendix to the preceding, we find a few points which demand a passing notice. In some cases, parents present no appearance of scrofula until some time after the disease has shown itself, or even proved fatal in their offspring. We do not controvert the fact, but must submit that, as a proof of the doctrine at which our author continually aims—the exclusively hereditary origin,—it is essentially inconclusive, for we are in no way enlightened as to the cause of the disease in the parents. For anything we know, the whole family may have been subjected to such external influences as, in the opinion of most pathologists, would suffice to develope scrofula, independently of any constitutional taint.

M. Lugol totally denies that scrofula ever passes over one generation, and reappears in the next. If this were the case, he says, a man would give what he does not possess—there would be effects without a cause. How, then, we would ask, do syphilitic persons and others produce scrofulous children, as he has endeavoured to show they do?

In an investigation of this nature, the observer must not always expect to find one cause alone the efficient agent; he will meet with many cases in which several have united their injurious influences. This was especially noticed in France, at the time when the conscription was in full operation, and our author draws a lamentable picture of the disastrous effects upon the nation at large which this mode of repairing the losses in the field produced. We must refer to the work itself for a fuller view of this subject, and also for an exemplification of the noxious effects not uncommonly produced upon scrofulous individuals by the excitements incidental to the married state. M. Lugol argues strongly, but we suspect without much chance of success, upon the expediency of a law to prevent the marriage of such as are affected with the disease in question.

Scrofula is peculiarly rife among the inmates of the foundling and orphan institutions of Paris. It is quite clear that, in the great majority of these cases, it is altogether impossible to adduce anything like evidence of hereditary transmission; while at the same time the external circumstances in which these miserable outcasts are placed are especially fitted to engender disease. True to his principle, however, M. Lugol, though admitting the influence of the latter in developing the affection, still affirms that it has been impressed upon these individuals from their birth, assuming that the parents were in such conditions as to render them incapable of having a sound progeny.

The subject next brought before our notice—viz. the production of scrofula in an originally healthy child, by using the milk of a scrofulous nurse—would, we think, find its more correct place in the succeeding portion of the work; for it is clearly the operation of an external influence. That the milk of a scrofulous woman should be unfit for the healthy nourishment of a child, is extremely probable; but we can see no analogy between this and the propagation of a disease by generation. In the one case the primitive elements of the future being are subjected to a morbid impression from the first moment of their receiving the vital impulse; in the other there is merely an imperfect elaboration of the materials destined to nourish a being already fully organized. The case, indeed, would be widely different, could we believe in the existence of a scrofulous *virus*, capable, like the syphilitic, of being propagated by infection; but all the results of experiment and observation are totally opposed to such an idea, as will be seen hereafter.

II. OF THE PATHOLOGICAL CAUSES OF SCROFULA. It has been frequently remarked, that the first development of scrofula can be dated from an attack of some other disease, and more especially of variola, rubeola, or hooping-cough; and many have regarded these as the real generating causes of the subsequent malady. We need scarcely say that M. Lugol utterly denies the correctness of such an opinion. In his view, they are merely the agents which call into activity an already existing predisposition. We have no intention of pronouncing any deliverance upon the point at issue; but we must record it as our decided opinion, that in the pages before us there is no *proof* whatever. It is not by bold assertions, and the narration of a few isolated cases, that a question of this nature can be determined to the satisfaction of an inquiring mind; and we appeal to all who may read the original, if we are presented with anything more than this. By far the greatest portion of the paragraphs allotted to the subject are occupied with an exemplification of what nobody denies, viz. that these diseases affect most severely and fatally those who possess a scrofulous constitution.

But there are other affections to which the same influence has been attributed; as mucous or catarrhal fever, the fever of growth, difficult dentition, intestinal worms, &c.—these M. Lugol regards, and probably with justice, not as causes, but as particular forms of the disease itself.

Again, when a woman becomes scrofulous during pregnancy, or after abortion or labour, it is merely the constitutional disease that is called into activity by these different states. Indeed he believes that the great majority of spontaneous abortions and of difficult labours are caused by the existence of the scrofulous taint.

*Erysipelas* is a frequent precursor of scrofula, especially of that form in which the skin and subcutaneous cellular tissue are chiefly affected. It is also a very common complication occasionally proving fatal, but most generally exerting a favorable influence upon the pre-existing disease. When occurring under these circumstances, its progress is usually tedious, and the favorable termination is always ushered in by the appearance of some critical evacuation, or of purulent collections in the glands or cellular tissue. In M. Lugol's opinion, it is never a *cause* of scrofula. May not its extreme frequency in the wards of St. Louis be in some measure connected with the iodine-treatment, to which it appears all the patients are subjected?

*Syphilis* and *gonorrhea* are among the worst complications of scrofula. They often light up the disease in those who are predisposed; they render it much more severe and intractable in its nature; and they frequently produce relapses in such as seemed to have been cured. Does M. Lugol regard these two affections as identical, that he includes them both under the one term, syphilis?

III. Of the external causes of scrofula. To the discussion of this subject, the third and concluding portion of the work, is devoted.

There are some localities where the disease prevails to such an extent, that it may be said to be *endemic.* Authors are much at variance as to the cause or causes of this, but humidity has been generally supposed to play an important part. Against this doctrine our author strenuously, and, it appears to us, successfully contends, by showing that the disease is endemic throughout provinces of considerable extent which are not damp, but have a dry climate; and that it does not prevail in many places which are extremely humid. Recent observation of the sort of antagonism between intermittent fever and tubercular disease of the lungs, also opposes such an idea. But while we are thus shut out from one explanation, the researches of M. Lugol, furnish us with no other; for though he severally accuses importation, the non-mercurial treatment of syphilis, and inter-marriage of relatives, as the cause of these local peculiarities, we must, in the absence of all proof, decline acknowledging these as the originating powers.

Yet, although humidity *alone* is thus inoperative, may it not, when acting in concert with other depressing agencies, such as variableness of climate, insufficient food and clothing, too severe, or too prolonged toil, bad air, &c., occasionally engender scrofula? To this also, M. Lugol replies in the negative; but we cannot regard his evidence as conclusive. For, though he narrates several cases which at first sight appeared to favour the affirmative but on a more rigorous examination proved to be merely examples of hereditary transmission; and though he affirms, certainly upon slender grounds, that scrofula is not common among the inmates of prisons, of camps, and of ships, who are often exposed to all these influences, we feel that something more is wanting, and that in the absence of official returns,* or of systematic personal investigations, carried on for a sufficient length of time, the *positive* results of other observers must weigh most heavily in the balance.

In like manner, and with equal looseness of argumentation, he affirms that *climate* can have no influence in the production of scrofula, because

* Are there no *medical officers* connected with the prison of Poissy who could have answered M. Lugol's inquiries regarding the prevalence of scrofula more satisfactorily than the one prisoner whom he consulted, or even than the person who was in the habit of employing the prisoners as his workmen? We receive such constant proofs of the ignorance of patients concerning their own diseases, that we place small confidence in their reports of the diseases of others.

the disease exists in all countries, and everywhere presents the same phenomena. At the same time, he acknowledges that the natives of tropical climates very commonly fall victims to strumous diseases, when brought into our temperate regions. Are we then to assume at once that all these had the congenital predisposition? That the monkeys and other animals continually dying in our menageries and zoological gardens, have all had scrofulous parents? and that Mr. Newport succeeded in tuberculizing his larvæ, only because these unfortunates shared in an hereditary taint? The idea is too ridiculous to be entertained for one moment.

The other supposed causes of scrofula, viz., vaccination, onanism, and conception during the menstrual period, may be passed by unnoticed. Nor is it necessary to do more than remark that we entirely agree with M. Lugol in his belief, that the disease with which we have been occupied is *non-contagious*, and that we most heartily join in his condemnation of the unwarrantable practice of inoculating healthy individuals with matter taken from diseased subjects.

We should observe, that of all the seasons of the year, spring is the most obnoxious to scrofulous subjects; the first attack of the disease is often developed at that time, and it is during the same period that relapses are most common. A knowledge of this fact may assist our prognosis, for when in the course of any case, it is observed that each spring exacerbation diminishes in severity, we may anticipate an eventual cure.

In regard to *treatment* we can say but little, for it is scarcely alluded to by the author; but from occasional passing remarks we can gather that *iodine* is still his sheet-anchor; and as no notice to the contrary is given, we presume the plan adopted now is the same which has been already for many years before the profession.

Our analytical task ends here, and it only remains for us to record our general opinion of the work with which we have been engaged. That opinion, we regret to say it, is not favorable. As a scientific production, the book is equally unworthy of M. Lugol's reputation, and of his position. We have examined it, as our readers will perceive, with care; but have failed to discover that he has thrown any new light upon the pathology of the disease, or developed any views that might have guided us to more successful treatment. And in regard to that one doctrine, in support of which his strength is so lavishly expended, the exclusively hereditary origin of scrofula, we remain still unconvinced. Freely conceding that it may have been often overlooked where it really did exist, and that in all probability it is more generally operative than many suppose, we should yet require much stronger proofs than any here adduced, ere we are led to banish all other causes from our thoughts. For reasons best known to himself, M. Lugol totally disregards the numerical method of investigation; in the whole volume there is not one calculation. And yet we are assured no question of this kind can ever be settled in any other way. Such vague expressions as *most, a large number, a few*, &c., well enough in ordinary conversation, and passable, perhaps, in academic prelections, are altogether out of place in a treatise that claims the title of 'Researches.' They carry conviction to none but those whose approbation is without value.

To one other thing we must refer, because, though bad at all times, it is peculiarly reprehensible in professional writings; we mean the tone of special pleading, which has so constantly jarred upon our feelings in the perusal of these pages. Trivial circumstances are magnified beyond due bounds, when by so doing the author's purpose might be served; and the very same are passed by without comment, when to have insisted on them would injure his argument. Take one example. Leucorrhea, uterine hemorrhages, &c. are included among the signs of scrofula, and a woman subject to these would, in other parts of the work, be accused of transmitting the disease from herself to her child; but when the same symptoms are mentioned as resulting from the mode of life of some females in Paris, (p. 154,) these females are not regarded as strumous, because if they were so, it would be an example of the generation of scrofula from external causes. One-sidedness of this kind is no less injudicious than unfair; the sole effect it can produce is to weaken our confidence in other statements, and cause us to regard with suspicion what may be really deserving of belief.

---

